# Axial stress versus strain responses of CFRP confined concrete containing electronic waste aggregates

**DOI:** 10.1038/s41598-023-50247-5

**Published:** 2023-12-27

**Authors:** Saad Ullah, Muhammad Irshad Qureshi, Panumas Saingam, Qudeer Hussain, Kaffayatullah Khan, Ekkachai Yooprasertchai

**Affiliations:** 1 Government College of Technology, Swabi, Pakistan; 2 Department of Civil Engineering, University of Engineering and Technology, Taxila, Pakistan; 3 Department of Civil Engineering, School of Engineering, King Mongkut’s Institute of Technology Ladkrabang, Bangkok, 10520 Thailand; 4 Dr. House Consultants Co. Ltd., Bangkok, Thailand; 5 Department of Civil and Environmental Engineering, College of Engineering, King Faisal University, Al-Hofuf, Kingdom of Saudi Arabia; 6 Department of Civil Engineering, Faculty of Engineering, Construction Innovations and Future Infrastructure Research Center (CIFIR), King Mongkut’s University of Technology Thonburi, Bangkok, 10140 Thailand

**Keywords:** Civil engineering, Mechanical properties, Composites

## Abstract

This research work investigates the axial stress versus strain responses of un-strengthened and carbon fiber reinforced polymer (CFRP) composites strengthened concrete specimens made with electronic waste coarse aggregates. For this purpose, 36 circular and non-circular 300 mm high concrete specimens constrained with CFRP sheets and partially replaced with E-waste coarse aggregates were prepared. The effect of cross-sectional geometry, 20% partial substitution of natural coarse aggregates with E-waste aggregates, corner effect of non-circular concrete specimens, confinement of specimens with CFRP sheets, and effect of the number of confinement sheets were also studied. In control concrete specimens, the coarse aggregates were 848 kg/m^3^ and E-waste aggregates were 212 kg/m^3^. The cement was 475 kg/m^3^ and fine aggregates were 655 kg/m^3^. Test results indicated that compressive strength is reduced by substituting natural coarse aggregates with E-waste aggregates. At the same time, compressive strength increased to 71%, 33%, and 25% for circular, square, and rectangular concrete specimens, respectively, by CFRP confinement. Whereas the axial strain increased to 1100%, 250%, and 133%, for circular, square, and rectangular concrete specimens, respectively, by CFRP confinement. CFRP sheets also enhanced the Poisson's ratio. Because of the greater confinement given by a double CFRP layer, it is more effective than a single layer. Furthermore, results also indicated that strength reduction in non-circular concrete specimens was greater than in circular concrete specimens for all studied cases. In the end, for theoretical calculations, strength and strain models for confined concrete suggested by different researchers were applied and compared with experimental results. In comparison to the experimental findings, theoretical data showed that most of the models were either on the higher or on the lower side, while only some model results matched well with the experimental data.

## Introduction

Electronic gadgets and machines are becoming increasingly common in people’s daily lives. Every household's electronic goods are expanding in number^1^. This development of electronic materials has improved human life in all major fields over the previous few decades. These materials have shaped enormous innovative improvements for human life according to the needs of the period, but electronic waste (E-waste) has now become an inescapable environmental concern^2^. Electronic waste, also called e-waste, are various forms of electric and electronic equipment that have ceased to be of value to their users or no longer satisfy their original purpose. Electronic waste (e-waste) products have exhausted their utility value through either redundancy, replacement, or breakage. Due to the lack of regulatory policies and informal E-waste processing, it can result in pollution and serious health issues. Current research focuses on the recycling of E-waste by-products from different sectors to effectively use them in concrete. Recycling of these waste materials helps to keep our environment healthy and sustainable^3–6^.

The waste generated by electronic products like monitors, laptops, LE/CDs, and televisions adds up to a significant amount of E-waste. The use of E-waste produces environmentally friendly, lightweight, and inexpensive concrete, but the decrease in strength due to E-waste limits its large-scale implementations in concrete. Furthermore, the need for aggregates in the concrete building sector is growing rapidly day by day, and by the end of 2025, it will increase up to 59%^7^. The natural resources for aggregates are depleting as a result of the continuous production of concrete products, which will have serious environmental consequences^8^. It is impossible to stop the manufacturing of electronics among common humanitarians in the current era of the internet and electronics. As a result, it is critical to take steps to recover reusable components from E-waste leftovers in order to ensure sustainable concrete development and to avoid environmental degradation^9,10^. Therefore, one of the possible strategies for creating a sustainable environment is to use E-waste aggregates in concrete. Aggregates from electronic waste are produced by heating them below their melting point. Electronic waste aggregates of particular sizes are then made by crushing of heated waste. It has been observed that strength is reduced by adding a specified amount of E-waste in concrete^11–16^. Therefore, the present study used carbon fiber reinforced polymer (CFRP) confinement to strengthen the specimens in order to improve the behavior and properties of E-waste concrete.

Some researchers studied the use of waste resources like E-waste^11,12,17–19^ and waste powder^20–22^ in concrete. Kumar et al.^1^ studied the effect of the replacement of E-waste and coconut shells up to 30% replacement of coarse aggregates. Results concluded that compressive strength and density of concrete decreased with the addition of these wastes. Akram et al.^23^ investigated the use of electronic waste as a partial replacement of coarse aggregates in concrete. Results concluded that cement cost is reduced due to the use of such waste materials. The use of electronic waste can help to reduce concrete's self-weight while simultaneously increasing its ductility. As a result, it improves concrete deformation before its failure. Alagusankareswari et al.^24^ performed experimental testing and statistical analysis on E-waste concrete. The application of load as a two-point on the specimen was the objective of the experimental research. Results concluded that the self-weight of concrete was decreased using E-waste. So, E-waste can produce lightweight concrete. On the other hand, partial substitution of coarse aggregates with E-waste results in the reduction of compressive, flexural, and splitting tensile strength. Shamili et al.^25^ studied E-waste concrete and concluded that the growing need for sand and gravel in the construction sector is due to the rapid development in the world population and widespread urbanization. It was concluded that adding E-waste to concrete produces lightweight concrete since E-waste reduces the weight of concrete because E-waste aggregates are lightweight and absorb less water than natural coarse aggregates. Compressive and flexural strength was also lower in the mix having E-waste as compared to the control mix. Iqbal et al.^26^ conducted research on Pakistan's rising issue related to electronic waste. The report describes the main facilities for electronic waste recycling, existing and prospective domestic electronic waste generation, electronic waste import, and various stages and obstacles in the country's electronic waste management. Anand and Hamdan^27^ studied the effect of the replacement of 20% of aggregates with E-waste on the compressive strength of concrete. Results concluded that compressive strength increased when 5% E-waste aggregates were used, while further increase in replacement decreased the compressive strength significantly. Further, mechanical properties of concrete made with recycled tire rubber were extensively studied in the past. It was observed that the strength of concrete mixes decreased with higher percentage replacement of fine aggregate by recycled tire rubber aggregates^28–31^. Various studies on concrete compression elements confined with CFRP sheets to improve structural behavior have been published in the literature^32–37^. Li et al.^34^ studied the use of CFRP jackets for the concrete produced with recycled aggregates from construction and demolition waste. In their study, total number 29 concrete specimens were cost and tested for both low and high strength concrete. It was found that the CFRP jackets are more useful for low strength concrete compared to the high strength concrete. Tang et al.^35^ also investigated the mechanical performance of CFRP confined geopolymeric recycled aggregate concrete. In their study, our aggregate replacement ratios (i.e., 0%, 25%, 50%, and 100%) and two thicknesses of CFRP jackets (i.e., 1 and 2 layers) were considered.

Although there are many studies on using fine plastic waste aggregate to produce lightweight concrete^12,16,18,19^, however, the axial stress versus strain responses of FRP-strengthened square concrete made with coarse plastic and or electronic waste (E-waste) aggregates are not yet explored. The use of E-waste coarse aggregates decreases the compressive strength, which must be enhanced for its practical implications. So, carbon fiber reinforced polymer (CFRP) composites strengthening will play a significant role in enhancing the properties of E-waste columns, which have not been studied previously. The tensile strength of carbon fiber-reinforced polymer composites is approximately 8–10 percent higher than that of glass fiber-reinforced polymer composites. Further, the carbon fiber handles more pressure than its traditional alternatives and survives the most severe environmental conditions like humidity, rainfall, radiation, and chemical exposure. In the past, extensive studies have shown that the use of CFRP composites is effective in enhancing the ultimate strength and strain of concrete. Therefore, the goal of this research is to look at the axial compressive performance of concrete specimens having E-waste and externally confined with CFRP composites. For this purpose, a total number of 36 specimens with circular, square, and rectangular cross-sectional forms were axially compressed and tested for various strengthening strategies. The effect of using E-waste aggregates, three different cross-sectional shapes, and the influence of CFRP single and double layer were studied for axial compressive strength, stress–strain behavior, Poison’s ratio, lateral strain, lateral dilation response, and failure patterns. In this way, this research work will provide environmentally friendly concrete as it conserves natural resources. Along with this, it reduces pollution, health issues associated with growing E-waste production, and its safe disposal.

## Experimental program

### Materials and their properties

**Figure 1:**
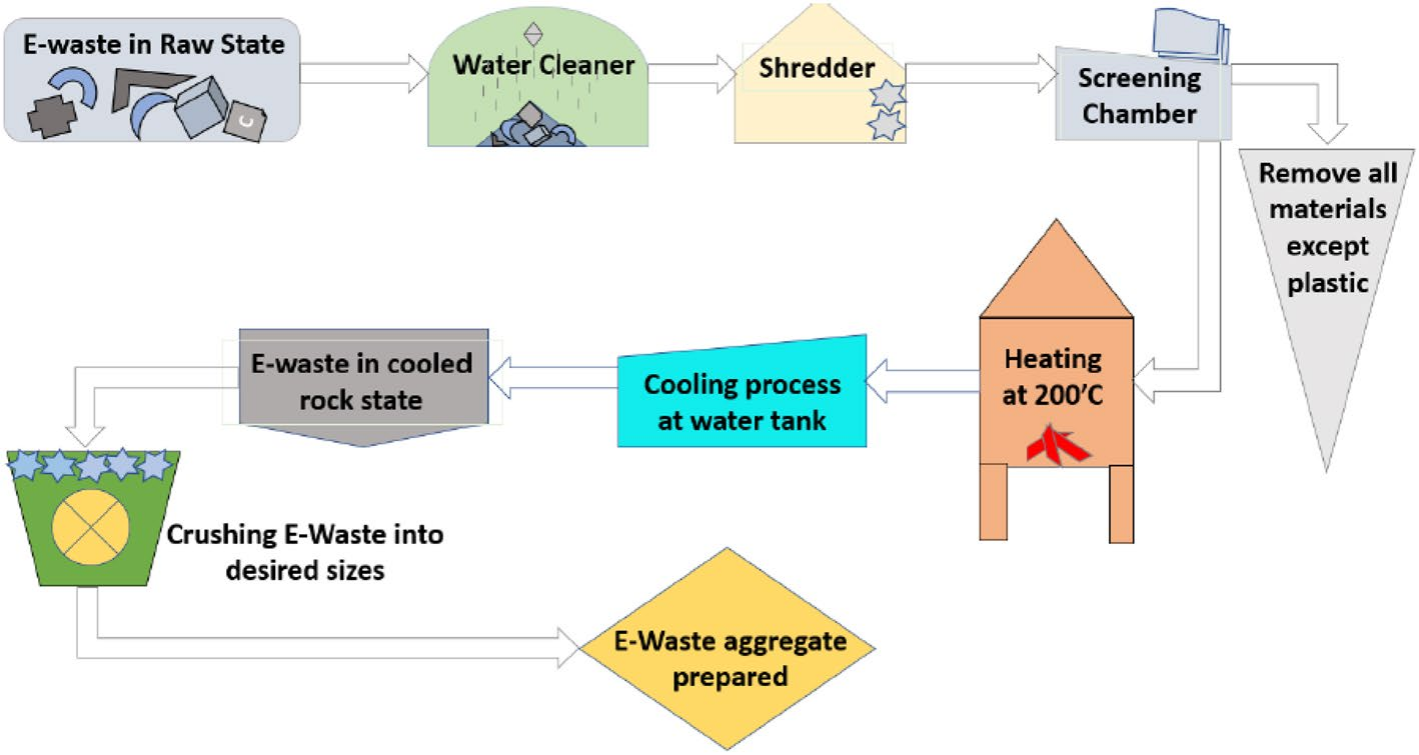
Production of E-waste aggregates.

**Figure 2:**
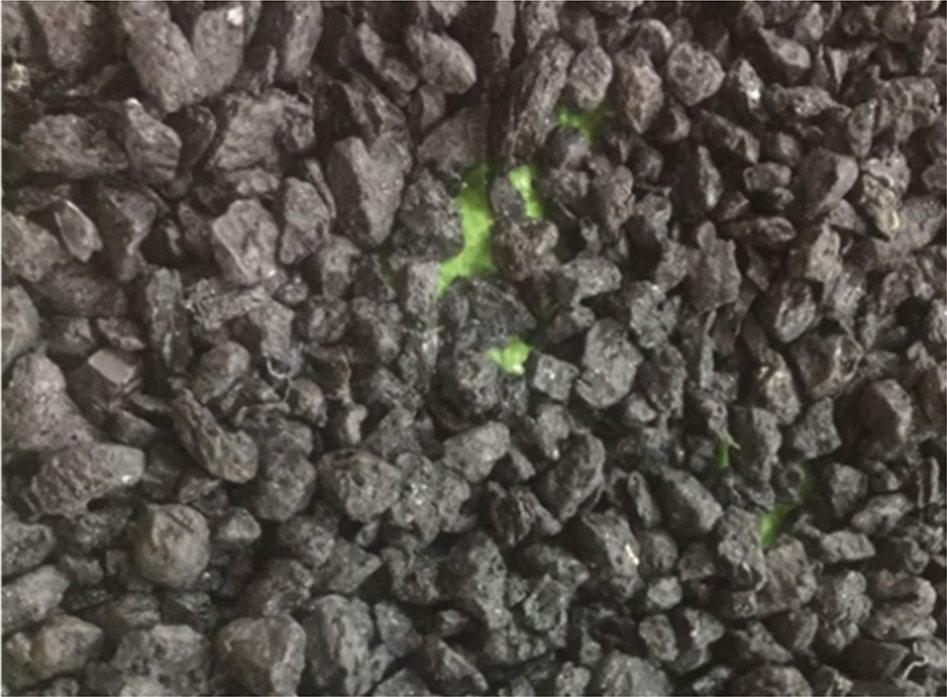
E-waste coarse aggregates obtained from electronic waste.

**Figure 3:**
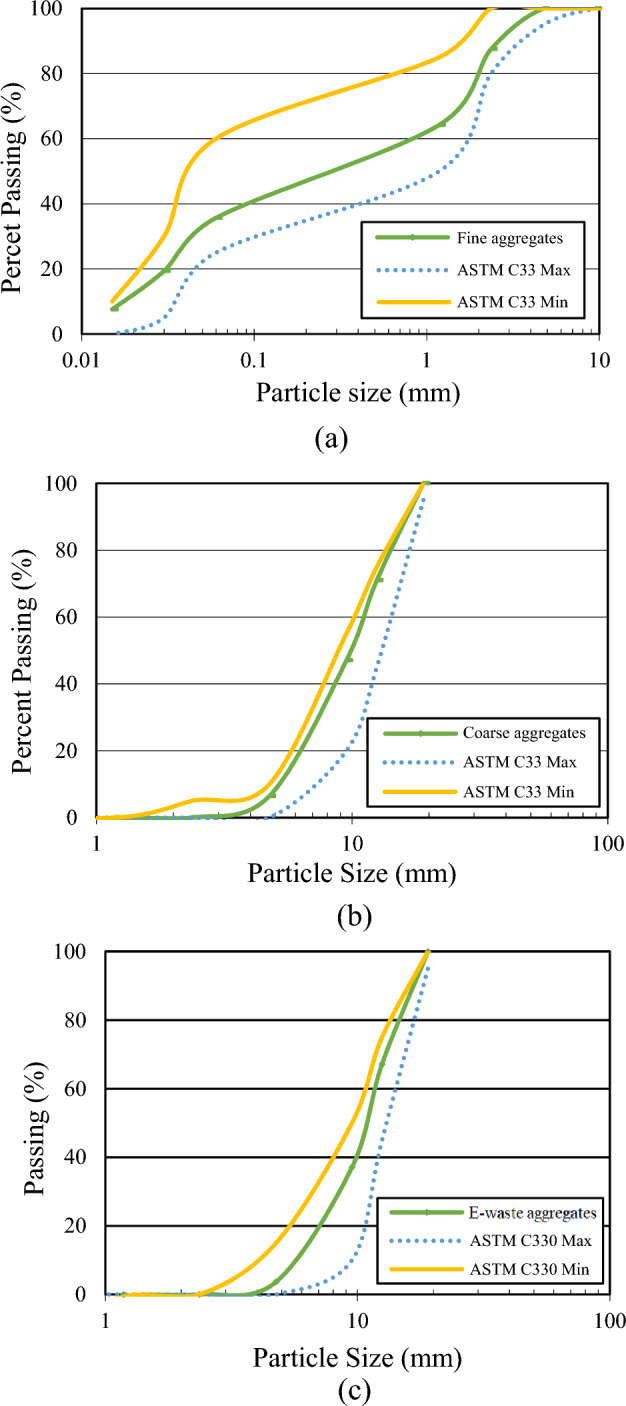
Particle size analysis of aggregates (**a**) fine aggregates, (**b**) coarse aggregates, (**c**) E-waste coarse aggregates.

Type-I Ordinary Portland cement was used to meet the standards of ASTM C150/C150M^38^. The electronic waste of computers was obtained from the local market. The obtained electronic waste is first softened in a kiln at a lower temperature than its melting point. The E-waste is then cooled and crushed into the desired sizes. Figure 1 shows the whole process of obtaining aggregates from electronic waste. In the screening chamber, all non-plastic materials, such as steel rings and pins, were manually removed. E-waste aggregates used in this study are shown in Fig. 2. Natural coarse aggregates with a maximum size of 12.5 mm were used, meeting the ASTM C33/C33M-18 specifications^39^. In the past, plastic aggregates of size 19 mm were found suitable to produce concrete, and the decrease in compressive strength was less than 20%^40^. Therefore, in this study, plastic aggregates of size 19 mm were used. The fine aggregates of Lawrancepur with a fineness modulus of 3.1 were used. In Pakistan, there are three sources of sand: Chenab, Lawrencepur, and Ravi rivers. Lawrencepur is a town in Attock District of Punjab Province in Pakistan. Lawrencepur Sand is known as top-quality sand in terms of well gradation as compared to Chenab and Ravi rivers. It is used in housing, commercial, and infrastructure projects all over Pakistan. The nearest source to this study was Lawrencepur. Therefore, in this study, finite aggregates were obtained from the Lawrencepur. Further, previous studies have shown that the fine aggregates of Lawrencepur could be effectively used in concrete Table 1 lists the properties of fine, coarse, and E-waste coarse aggregates, and their granular analysis is shown in Fig. 3. To achieve a uniform concrete mix, the superplasticizer Sika ViscoCrete®-3425 (aqueous solution of modified Polycarboxylates was in the form of clear liquid) was used.

**Table 1:** Properties of fine, coarse and E-waste coarse aggregates.

Property	Fine aggregates	Coarse aggregates	E-waste coarse aggregates
Water absorption (%)	0.78	1.35	–
Dry density (kg/m^3^)	1560	1520	535
Specific gravity	2.45	2.58	1.01
Fineness modulus	2.9	7.40	7.54
Maximum size (mm)	4.75	12.5	19
Minimum size (mm)	–	4.75	4.75

### Specimens and mix proportion detail

In this study, a total number of 12 concrete batches were made. The details of mix proportions are given in Table 2. The mix properties were designed in such a way as to achieve 28 days strength of 40 MPa for circular specimens (diameter = 150 and height = 300 mm) for control specimens. However, the actual strength was slightly higher than the designed strength. Three specimens were cast for each batch. Table 2 lists the specific designations for each batch. Since the water absorption of E-waste aggregates is almost zero as compared to the natural coarse aggregates, in this study, a water-to-cement ratio of 0.5 was used to avoid high slump and or workability of E-waste aggregate concrete. Previously, Ali et al.^11^ studied the effect of E-waste aggregate replacement in concrete. In their study, the E-waste aggregates were replaced by 10, 15 and 20%. The highest decrease in compressive strength was observed for 20% replacement. Therefore, in this study, 20% replacement of E-waste aggregates was considered. The letters C stand for circular, S for square, and R for rectangular specimens. 20E-WC stands for 20% electronic waste coarse aggregates. L0 is used for samples without CFRP confinement. L1 and LII stand for single and double layers of CFRP confinement on the specimens.

**Table 2:** Mix proportions and specimen labels.

Specimen labels	Cement (Kg/m^3^)	Coarse aggregates (Kg/m^3^)	Coarse E-waste (Kg/m^3^)	Fine aggregates (Kg/m^3^)	Plasticizer (Kg/m^3^)	Water (Kg/m^3^)	CFRP sheets
CCTRL	475	1060	0	655	3.1	237.5	0
C20E-WCL0	475	848	212	655	3.1	237.5	0
C20E-WCL1	475	848	212	655	3.1	237.5	1
C20E-WCLII	475	848	212	655	3.1	237.5	2
SCTRL	475	1060	0	655	3.1	237.5	0
S20E-WCL0	475	848	212	655	3.1	237.5	0
S20E-WCL1	475	848	212	655	3.1	237.5	1
S20E-WCLII	475	848	212	655	3.1	237.5	2
RCTRL	475	1060	0	655	3.1	237.5	0
R20E-WC L0	475	848	212	655	3.1	237.5	0
R20E-WCL1	475	848	212	655	3.1	237.5	1
R20E-WCLII	475	848	212	655	3.1	237.5	2

**Figure 4:**
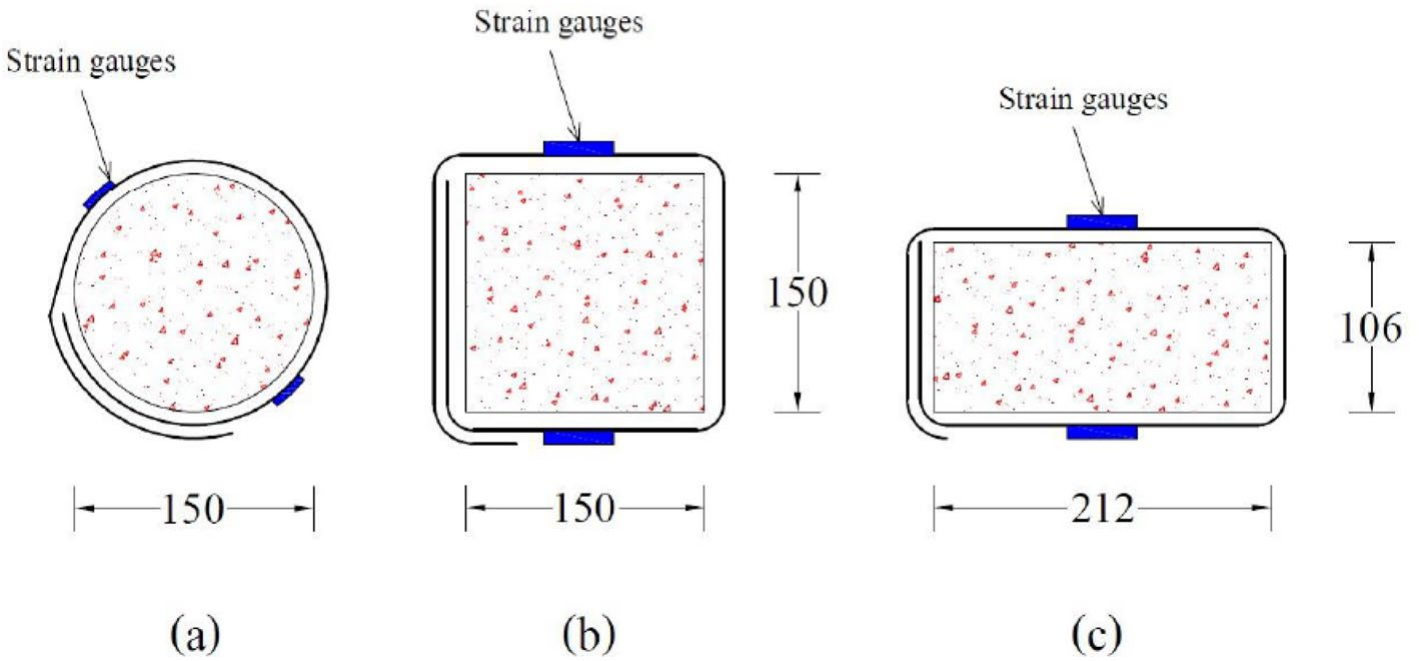
Cross-sectional dimensions of specimens, CFRP overlap positions, and strain gauge locations on the samples (units in mm).

For three different geometries, 36 specimens were cast, as shown in Fig. 4. Circular specimens have a diameter of 150 mm. Rectangular and square samples had cross-sectional measurements of 150 mm × 150 mm and 212 × 106 mm, respectively. The casting process was broken down into three parts. In the first stage, control samples were made without confinement and E-waste. After that, in circular, square, and rectangular specimens, 20% E-waste coarse aggregates and 80% natural coarse aggregates were added. In the third stage, samples for each mix and geometry were made as in the second stage, and then single and double layers of CFRP confinement were applied with a 150-mm overlap to strengthen the samples cast by replacing natural coarse aggregates with E-waste aggregates.

### Casting and curing procedure

**Figure 5:**
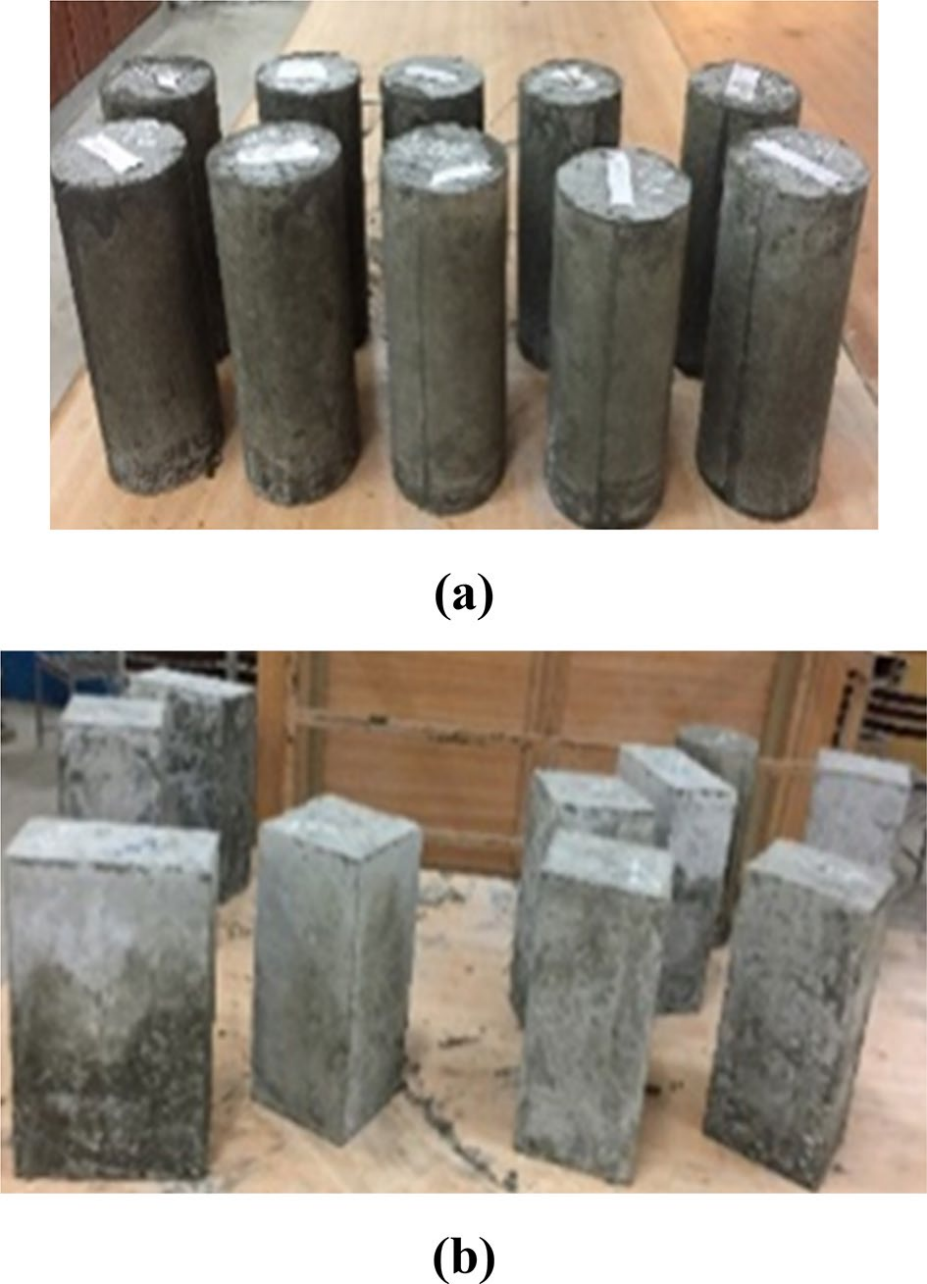
(**a**) Circular specimens after demolding; (**b**) square and rectangular specimens after demolding.

Molds of steel were used to cast circular specimens 300 mm in height and 150 mm in diameter. An elimination board was used for the remaining sections. Before pouring the concrete into the circular, square, and rectangular molds, the joints were tightened. The mix was made in the laboratory using a machine mixer with a capacity of 0.15 m^3^ and a rotational speed of 20 revolutions per minute. Firstly, the mixing of fine and coarse aggregates was done. Then water and cement were added, and further mixing was done. Figure 5 shows the circular, square, and rectangular casted specimens.

### Confinement of concrete specimens with CFRP

Previous research has shown that adding E-waste aggregates to concrete reduces its strength^12,16,18,19^. Therefore, in the current experimental work, CFRP confinement (Wrap Hex -230 C) was chosen to improve the strength and performance of E-waste concrete. Sandpaper was used to clean the surface of specimens on which CFRP was applied. For the removal of dust particles, the cleaned surface was thoroughly polished with cotton. The properties of CFRP sheets are listed in Table 3 as taken from the manufacturer.

**Table 3:** Different parameters of CFRP sheets.

Parameter	Value
Width of fabric	300–600 mm
Length/roll	≥ 50 m
Tensile elastic modulus	231, 000 N/mm^2^
Density	1.78 g/cm^3^
Tensile strength	4100 N/mm^2^
Strain	1.7%
Thickness	0.12 mm
Areal weight	220 g/m^2^ ± 10 g/m^2^

**Figure 6:**
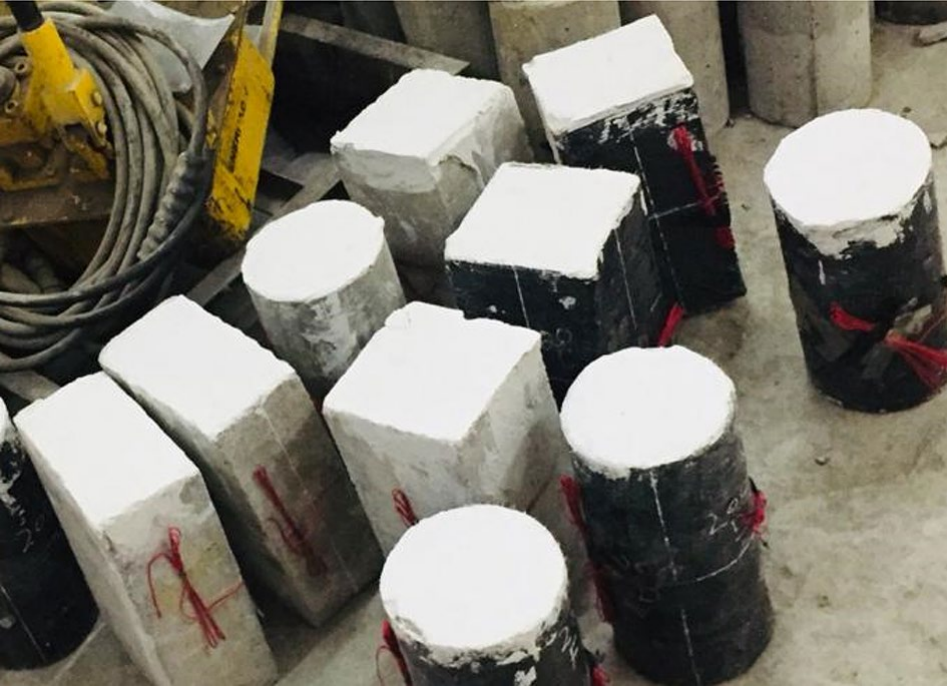
CFRP specimens.

Required sizes of CFRP sheets with an overlap of 150 mm were prepared carefully for each specimen. The epoxy chemical mix produces a jelly-like product that is dangerous for the skin, so the application of epoxy is done by using gloves on the hands. Composite specimens perform identically due to adhesion. Sikadur®-330 epoxy glue, with tensile elastic modulus, ultimate tensile strain, and tensile strength of 4500 MPa, 0.9%, and 30 MPa, respectively, was used to adhere the CFRP sheets to the samples. Figure 6 shows CFRP sheets being applied to prepared samples. For uniform distribution of load, Sulphur capping was also done before testing to eliminate small surface irregularities that occurred during casting.

### Experimental setup

**Figure 7:**
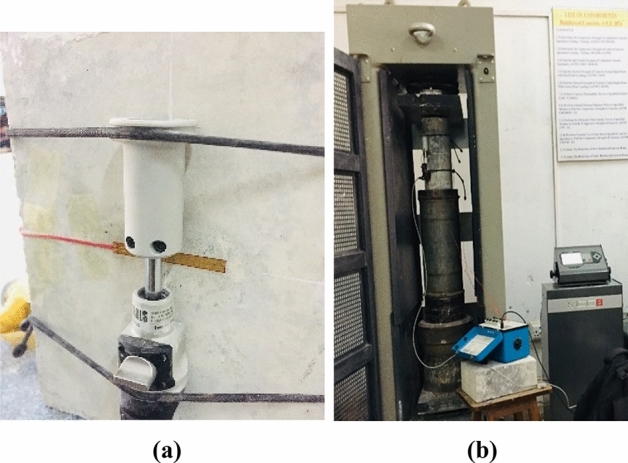
Experimental setup (**a**) instrumentation, (**b**) compression testing.

Two linear strain gauges that had a gauge length of 60 mm, 180° apart in opposite directions to each other, were attached at mid-height of the specimen for measuring lateral strain. Furthermore, three extensometers with a gauge length of 50 mm, at an angle of 120° to each other, were put in mid-height for measuring axial strain. For CFRP-confined samples, lateral strain was calculated using KFGS-30, whereas lateral strain for unconfined samples was calculated using kc-60. As a safety precaution, linear variable differential transducers having 150 mm gauge length were also mounted to monitor the axial strain. A load-controlled universal testing machine (UTM) with a maximum load of 5000 kN was used for testing samples. To apply preload, compressive load at a rate of 0.06 MPa/sec was applied. Then, until failure, the service load was applied at a rate of 0.030 MPa/s. Figure 7 shows the specimens' instrumentation and testing.

## Results and discussion

### Effect of coarse aggregates replacement

**Figure 8:**
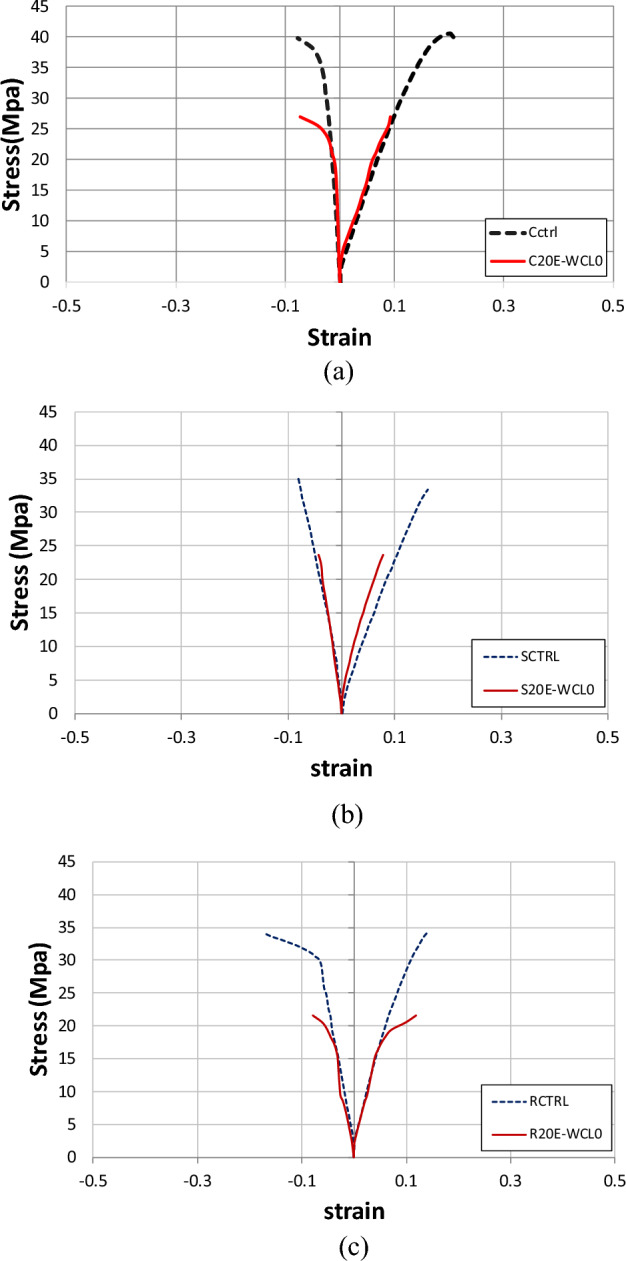
Stress–strain response of (**a**) control and E-waste circular samples, (**b**) control and E-waste square samples, and (**c**) control and E-waste rectangular samples.

Figure 8 shows the axial stress–strain behavior of circular, square, and rectangular control specimens, as well as specimens containing 20% E-waste aggregates. The average results of three samples were plotted to ensure more reliability. In the case of unconfined specimens, strain gauges were mounted on the surface of specimens. As a result, vertical cracks were found in these samples at peak load. These cracks in concrete primarily affected the strain gauges. For CFRP-constrained samples, strain gauges remained unaffected by concrete cracks, and damage in strain gauges occurred because of the CFRP sheet’s rupture due to its tensile failure. The tensile failure of CFRP sheets is mainly due to the lateral outward expansion of the concrete. The stress–strain performance of specimens having natural coarse aggregates is better than 20% E-waste coarse aggregate samples, demonstrating that compressive strength decreases by replacing the natural coarse aggregates with E-waste. As stated in previous research, the hydrophobic character and weak bonding of E-waste in the matrix resulted in compressive strength reduction^11–14^. It is also indicated in the literature that E-waste replacement resulted in lower stress values as compared to control specimens; hence, confinement is required^12,14,16^.

### Axial stress–strain behavior of CFRP confined samples

**Figure 9:**
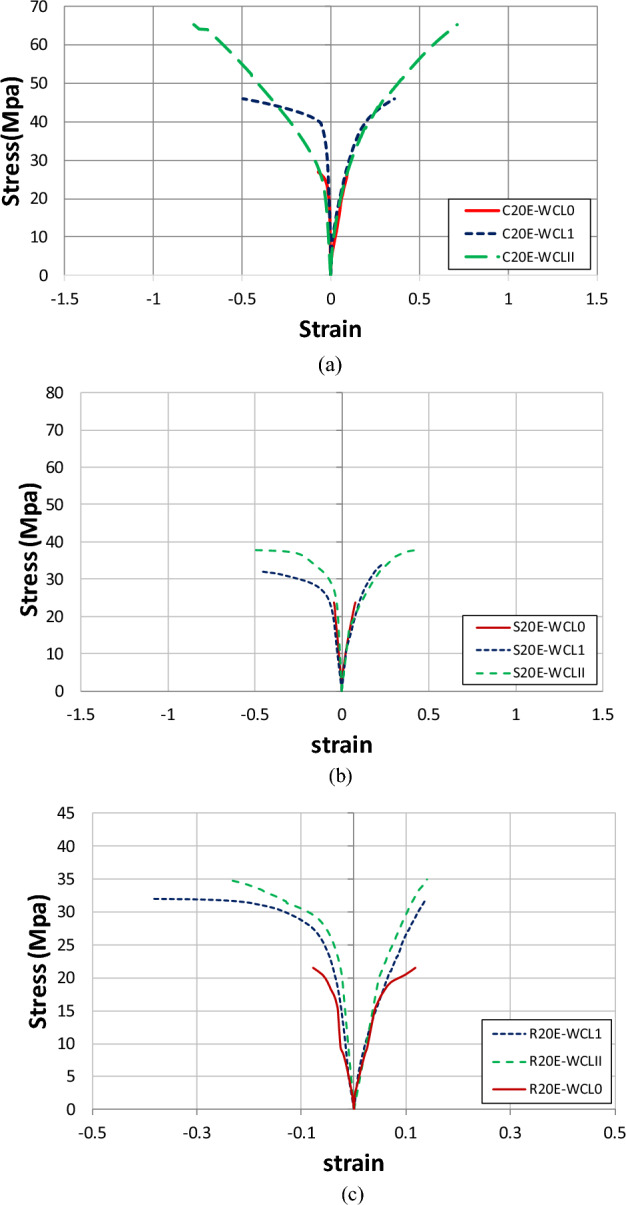
Stress–strain behavior of (**a**) confined and unconfined circular samples, (**b**) confined and unconfined square samples, and (**c**) confined and unconfined rectangular samples.

Figure 9 shows the axial stress–strain behavior of circular, square, and rectangular samples with and without CFRP confinement. Peak strain, stress, and failure behavior have all been proven to be significantly affected by CFRP confinement. The specimens without CFRP exhibited linear behavior and failed after reaching peak strength, whereas specimens with CFRP layers exhibited bilinear behavior. The first part of the curve is described by a linear line similar to the curve of the unconfined concrete. In the second part, stress–strain curves of confined concrete increase linearly again but with a much lower elastic modulus compared to the first part. The ultimate stress is also increased by CFRP confinement. When compared to the control specimens, the stress–strain response of confined specimens showed significant improvements in axial stress and strain, indicating an increase in stiffness and ductility due to single and double layers of CFRP sheets. Improvement in axial strength, stiffness, and ductility due to CFRP confinement is also mentioned in previous research^41^. When all three shapes were compared, circular samples showed a better performance. In comparison to all other specimens, including the control specimens, the circular specimen C20E-WCLII displayed the greatest values of stress versus axial strain. The reason for this could be the sharp corners of square and rectangular shapes, which causes CFRP sheets to fail and, ultimately, specimens to fail. To prevent CFRP sheets and specimens from failing prematurely, corners should be rounded for rectangular and square samples. Additional quantitative analysis is presented in section “CFRP confinement effect on axial strength and axial strain”.

### Poisson’s ratio vs. stress and strain behavior

Poisson's ratio is one of the important parameters for finding the effectiveness of lateral confinement. The ratio of lateral to longitudinal strain in concrete is called Poisson's ratio. Table 4 shows the ultimate Poisson's ratio value for all samples. The static Poisson's ratio normally for hardened concrete varies between 0.15 and 0.25^42^. However, in this study, the Poisson’s ratio was slightly higher due to the use of extensometers for axial strains. Further, for most of the specimens, with a 60–70 percent increase in compressive strength, Poisson's ratio decreases steadily and then increases with the further increase of axial stress.

**Table 4:** Poisson’s ratio value.

Sample	Ultimate value of PR	% increase in PR
CCTRL	0.42	–
C20E-WCL0	0.79	88
C20E-WCL1	1.38	229
C20E-WCLII	1.09	160
SCTRL	0.55	–
S20E-WCL0	0.56	2
S20E-WCL1	2.45	345
S20E-WCLII	1.31	138
RCTRL	0.45	–
R20E-WCL0	0.81	80
R20E-WCL1	2.80	522
R20E-WCLII	1.73	284

**Figure 10:**
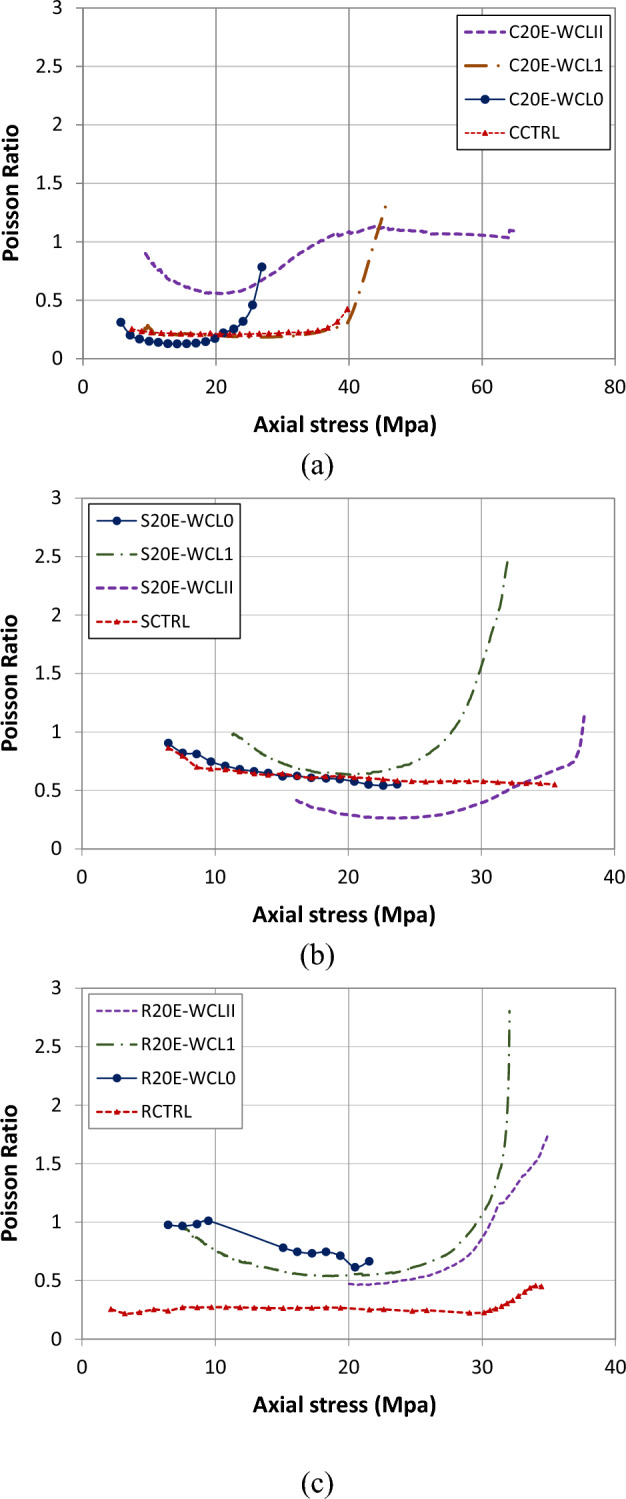
Poisson’s ratio vs. axial stress of (**a**) circular specimens, (**b**) square specimens and (**c**) rectangular specimens.

**Figure 11:**
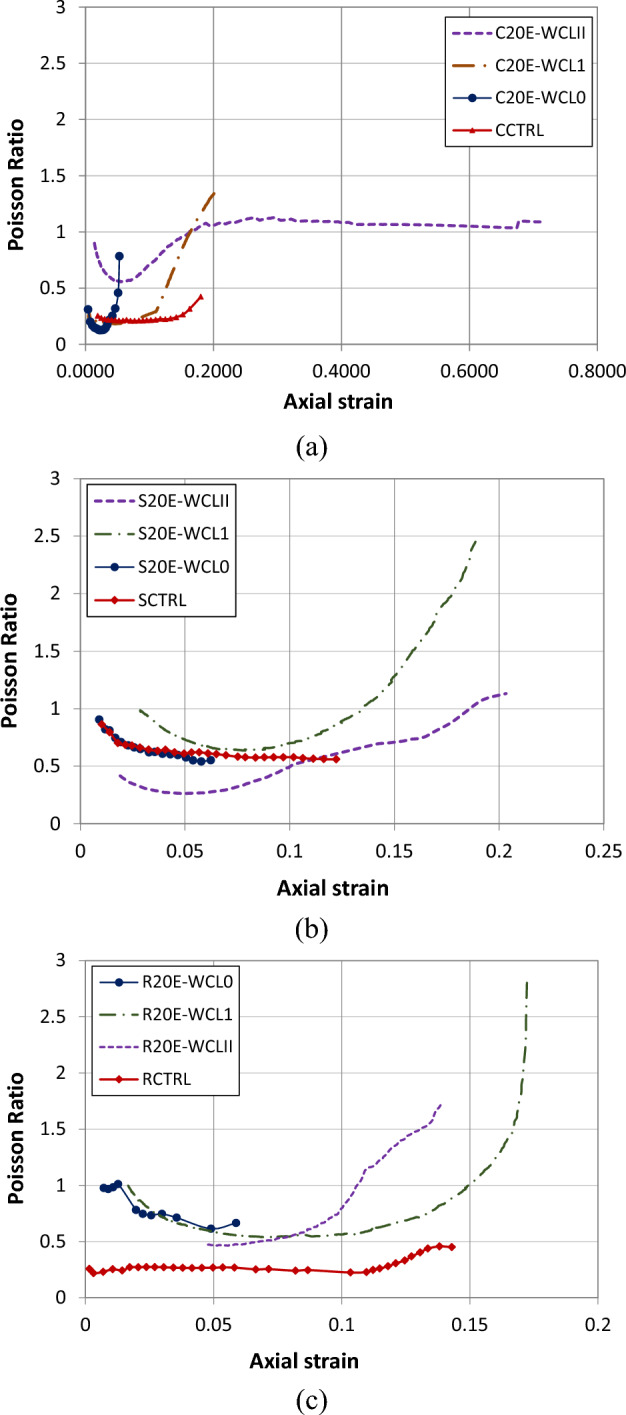
Poisson’s ratio vs. axial strain of (**a**) circular specimens, (**b**) square specimens and (**c**) rectangular specimens.

The Poisson’s ratio vs. axial stress and strain behavior of circular samples are shown in Figs. 10a and 11a. In circular specimens, specimen CCTRL has the lowest value of 0.42, while specimen C20E-WCL1 has the highest value of Poisson’s ratio of 1.38 (increment of 229%). Figure 10a shows that replacing E-waste with natural coarse aggregates enhances the Poisson’s ratio. From the graphs of circular, square, and rectangular specimens, it can be seen that the graph is straight initially, showing the constant value of Poisson’s ratio, whereas, after that, lateral strain increases significantly, leading to an increase in Poisson's ratio value. The response of CFRP confinement on the Poisson's ratio for circular samples is shown in Fig. 11a. As can be seen from the graph, the specimen with a double layer of CFRP confinement has significantly higher values of axial strain and stress and lower values of Poisson’s ratio than the single-layer specimen. Moreover, single-layered CFRP confined circular samples exhibited a greater value of Poisson’s ratio at failure, indicating that double layers of CFRP confinement are more effective than a single layer. The enhanced Poison’s ratio of samples can be attributed to effective lateral CFRP confinement^11^. The effect of CFRP confinement on Poisson's ratio value of square samples is depicted in Fig. 10b. The sample S20E-WFL0 has the lowest Poison's ratio value of 0.55, while the specimen S20E-WCL1 has the highest Poison's ratio of 2.45 (345% increase). Similar to the circular samples, square samples with double CFRP confinement layers have significantly higher axial stress and strain values and less value of Poisson's ratios than single layer specimens, showing that double CFRP confinement is also more effective for square specimens than single layer. Similarly, for rectangular specimens (Fig. 11b), the control (RCTRL) specimen has the lowest Poison’s ratio of 0.45, while the specimen R20E-WCL1 has the highest Poison's ratio of 2.8 (522% increase). Figure 10c shows that replacing coarse aggregates with E-waste increased the value of Poisson’s ratio. Because rectangular specimens have more sharp edges, the effect of replacement is higher. Figure 11c shows the effect of CFRP confinement on the Poisson's ratio for rectangular samples. Results concluded that samples with a single CFRP layer have a greater ultimate Poisson’s ratio value than the double-layer specimen. Two layers of CFRP demonstrate a substantial confinement effect, with Poisson’s ratio initially decreasing because of the confinement effect and then increasing before failure. Specimen with a single layer, on the other hand, has a decreasing value of Poisson’s ratio initially and then increases rapidly as the stress increases, and finally, it reaches its maximum value just before failure. In general, lateral confinement increased the Poison's ratio response significantly. From the results, it can also be concluded that circular samples have a higher value of axial strain as compared to square and rectangular samples, implying that circular specimens performed better throughout the confinement process. The use of two CFRP sheets for lateral confinement improves both the axial and lateral stresses of the specimens. It is also mentioned in the previous literature that^43,44^ CFRP sheet enhances the axial and lateral stresses of specimens, and these specimens have higher ductility and axial stiffness. So, confinement by CFRP sheets greatly improves the strength, stiffness, and ductility of concrete having E-waste coarse aggregates.

### CFRP confinement effect on axial strength and axial strain

**Figure 12:**
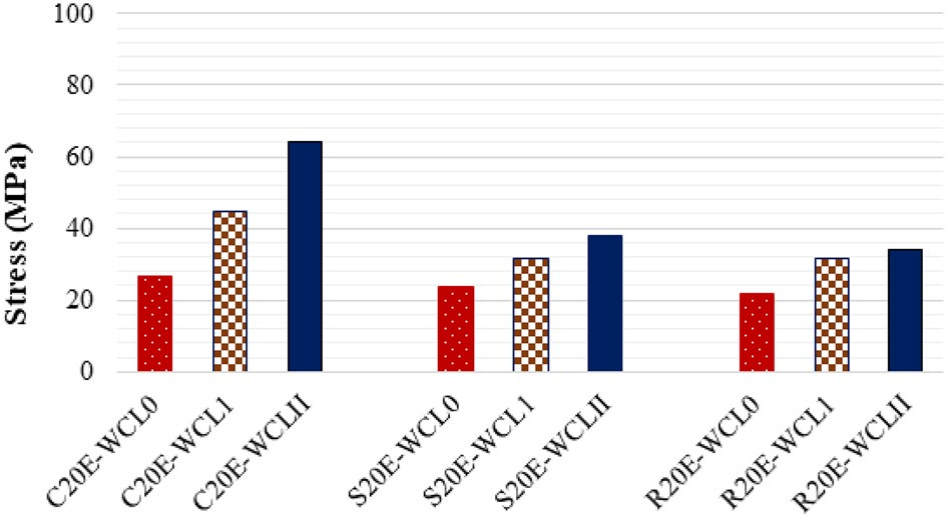
CFRP confinement effect on axial stress.

The compressive strength of samples having E-waste with and without confinement are compared in Fig. 12. The graph showed that compressive strength is enhanced by CFRP confinement, and double CFRP confinement enhances the compressive strength greater than that of single CFRP confinement. The specimen C20E-WCLII has the maximum axial compressive strength, with a value of 64 MPa, which is 137% greater than the C20E-WCL0 specimen. Single CFRP confinement increased the axial strength of samples C20E-WCL1, S20E-WCL1, and R20E-WCL1 by 67%, 34%, and 46%, respectively, in respect to their samples without CFRP. In the same way, the double CFRP confinement increases the axial strength of samples C20E-WCLII_,_ S20E-WCLII, and R20E-WCLII by 137%, 58%, and 55%, respectively, with respect to the corresponding samples without CFRP. These results indicate that the double layer is more effective than the single CFRP layer because the double layer provides more confinement.

**Figure 13:**
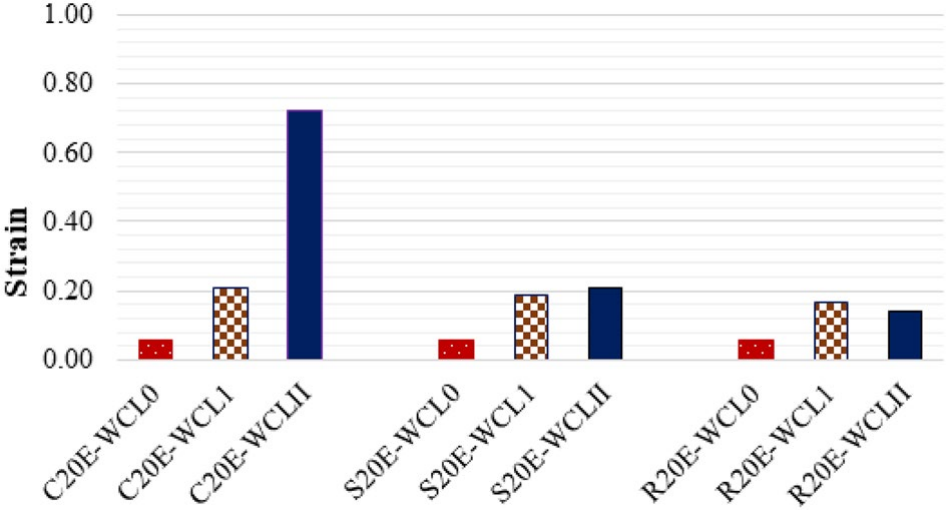
CFRP confinement effect on axial strain.

The ultimate axial strain of samples having E-waste with and without confinement are compared in Fig. 13. The graph shows that CFRP confinement enhances the ultimate axial strain for all samples and that a double layer of CFRP increases compressive strength more than a single layer of CFRP. Because of the sharp corners of rectangular and square concrete specimens, the ultimate strain for circular specimens increases more than that of square and rectangular samples. Sharp corners resulted in CFRP sheet failure and, eventually, specimen failure. The specimen C20E-WCLII has the largest ultimate axial strain, which was 1100% higher than the C20E-WCL0. In comparison to their samples without CFRP, the single CFRP confinement has enhanced the ultimate axial strain of samples C20E-WCL1, S20E-WCL1, and R20E-WCL1 by 250%, 217%, and 183%, respectively. In the same way, double CFRP confinement enhanced the ultimate axial strain of samples C20E-WCLII_,_ S20E-WCLII, and R20E-WCLII by 1100%, 250%, and 133%, respectively, when compared to samples without CFRP. As a result, a double CFRP layer is more effective than a single layer because the double layer provides more confinement. These findings revealed that confinement of E-waste concrete specimens by CFRP improves the axial strength, axial stiffness, and ductility, as also described in the literature^43,44^.

### Patterns of failure

**Figure 14:**
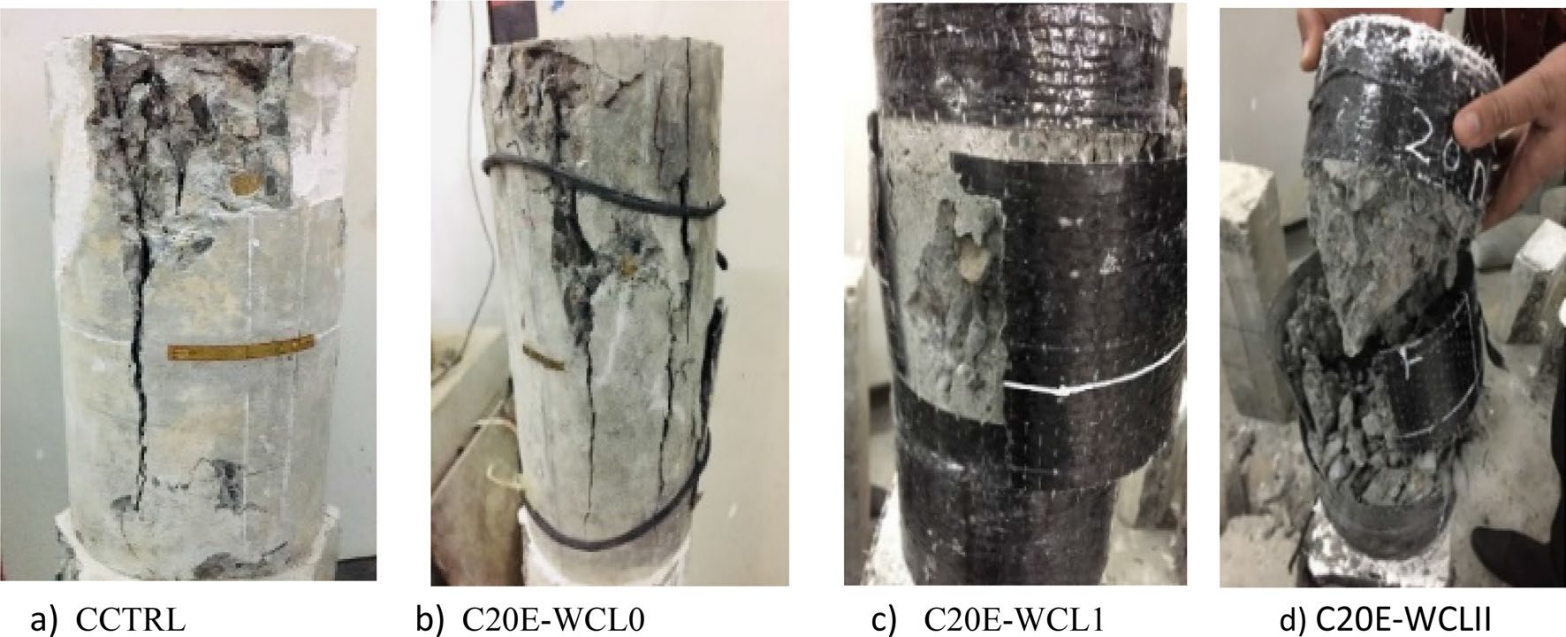
Patterns of failure in circular samples.

Patterns of failure of circular samples are shown in Fig. 14. Linear increment in stress and strain was shown by circular samples when the axial compressive load was applied up to the specimens' ultimate strength. When compared to the samples with E-waste aggregates, the control samples, without confinement and E-waste aggregates, had a higher tendency to rupture and readily split into two pieces with wider gaps. As evidenced by the literature^2^, the samples with E-waste aggregates have a higher tendency of crack arresting. CFRP sheets were ruptured after achieving their ultimate tensile strength, causing the specimens to fail. Prior to the ultimate failures, the de-bonding of the CFRP was not observed. The samples having E-waste aggregates have higher strain as compared to control samples, which shows that E-waste samples have a higher ductility. During failure, circular samples without confinement displayed cracks in the vertical direction, whereas rupture of the CFRP sheet in the central region was shown by samples having CFRP confinement. Because of the lighter weight of E-waste aggregates, the suggested concrete having E-waste and CFRP confinement could be employed in situations where lightweight concrete is needed without compromising the strength^2^.

**Figure 15:**
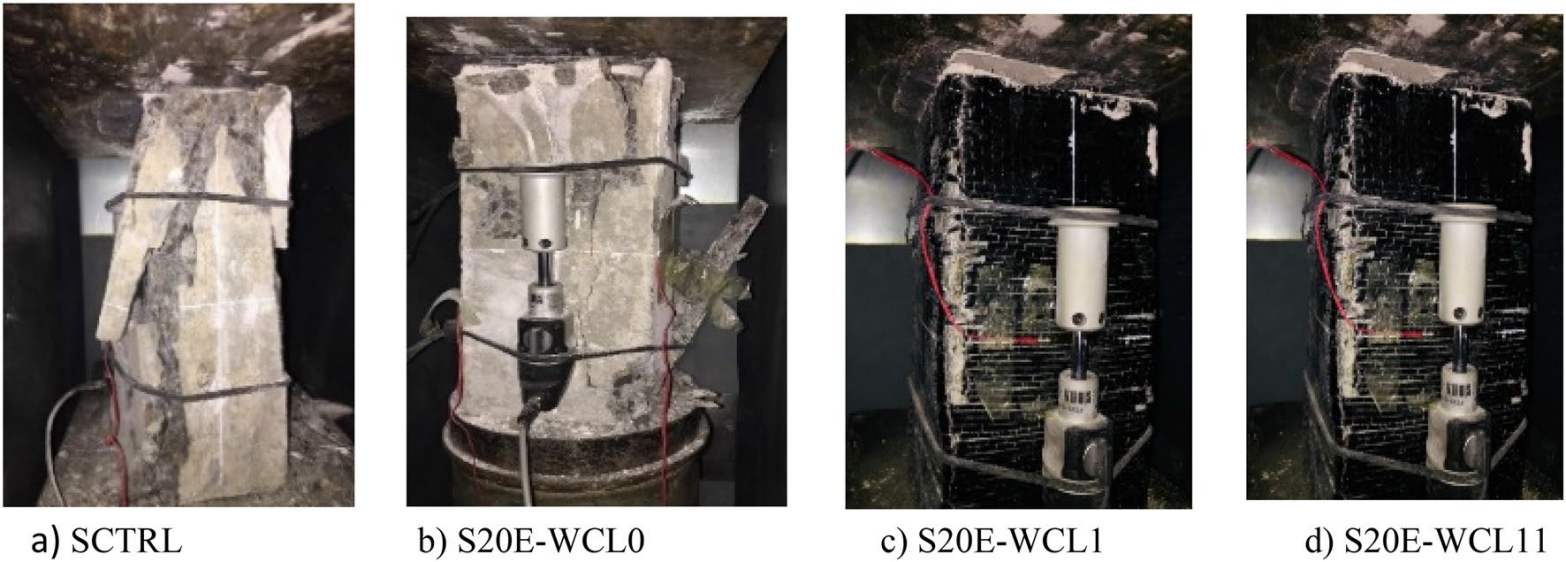
Patterns of failure in square samples.

Patterns of failure in square samples are shown in Fig. 15. Up to 60–70% of the ultimate load, linear increment in stress and strain was showed by square samples. After that, strain increased rapidly, ultimately leading to tensile failure of CFRP confinement. When compared to samples with E-waste aggregates, the control samples, without confinement and E-waste aggregates, had a higher tendency to rupture and readily split into two pieces with wider gaps. This is owing to E-waste aggregates, which have a larger crack-arresting potential, as mentioned in the literature^2,15^. In most of the confined square samples, CFRP sheets burst around the corners of samples when the ultimate tensile strength of the CFRP sheet reached, causing the specimens to fail.

**Figure 16:**
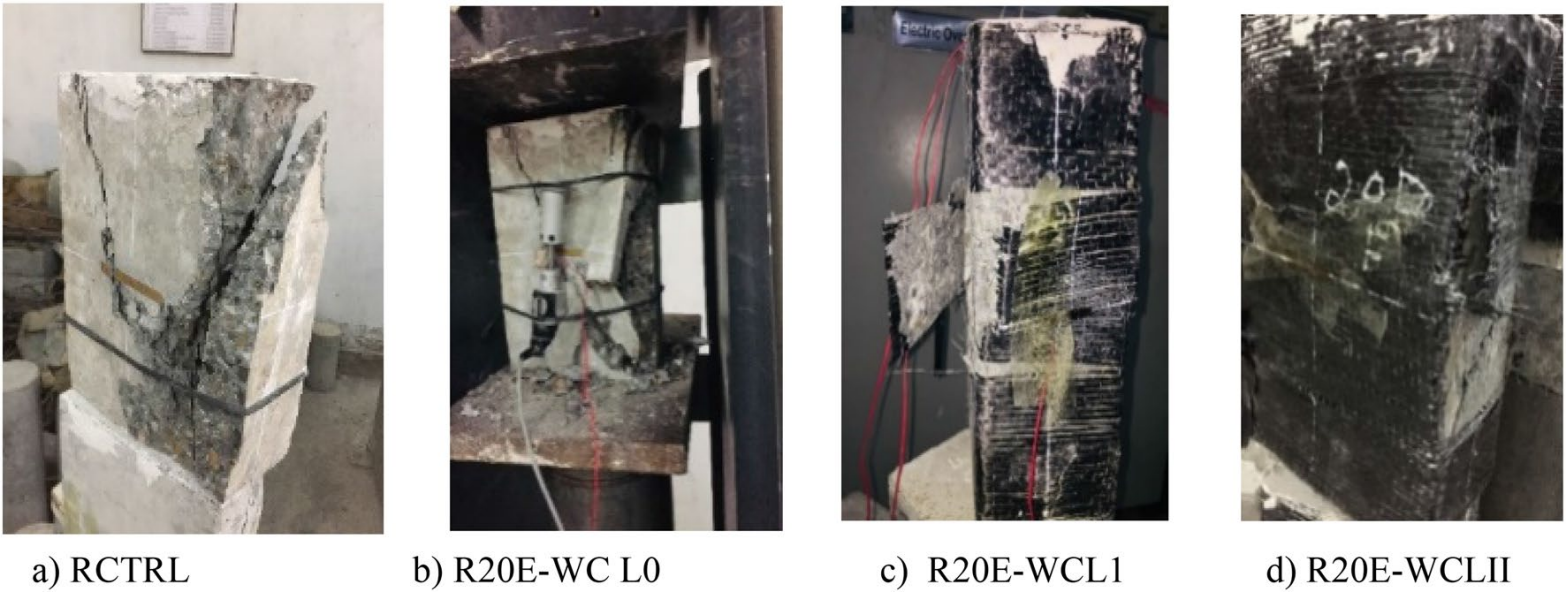
Patterns of failure in rectangular samples.

Patterns of failure in rectangular samples are shown in Fig. 16. Up to 60–70% of the ultimate load, linear increment in stress and strain was showed by rectangular samples. After that, strain increased rapidly, ultimately leading to tensile failure of CFRP confinement. Due to a higher crack arresting potential, the control rectangle specimen RCTRL, like the circular and square specimens, showed more rupture tendency in comparison to E-waste aggregates samples^15,45^. The rectangular concrete specimens exhibited the lowest strength with respect to other shapes because of sharp edges and asymmetrical geometry, due to which the CFRP sheet ruptured around the sharp edges of samples. It is also mentioned in the literature that rupturing of CFRP confinement around the specimens' corners causes the confinement and specimens to collapse prematurely^46^. In the past, similar failure modes have also been observed for CFRP-confined concrete made with different types of aggregates, such as natural aggregates, brick waste aggregates, and construction and demolition waste^47,48^.

## Detail of existing analytical models

In recent years, a wide range of analytical investigations has been carried out in order to establish models for the prediction of different properties of rectangular, square, and circular specimens constrained by unidirectional CFRP. There are several models developed for determining the theoretical values of stress and strain of CFRP-confined specimens. Table 5 shows a few models for three different shapes that have been identified in the literature^49–56^. Some researchers have developed models for all three shapes studied in this research work. Shehata et al.^55^ and Cusson and Paultre^56^ studied square and rectangular concrete specimens and provided models for these two shapes only, whereas Candappa et al.^49^, Lam and Teng^50^, and Lu and Hsu^51^ focused on the confinement effect on circular concrete specimens and provided models for circular shape concrete specimens. Almost all of the models developed for FRP-confined concrete can be represented as follows:

$$\frac{{f_{cc} }}{{f_{co} }} = 1 + k_{1} \left( {\frac{{f_{l} }}{{f_{co} }}} \right)$$

**Table 5:** Existing stress and strain models.

Study	Equation for stress	Equation for strain	Sample shape
Candappa et al. 2001^49^	$f_{cc} = \left[ {1 + 5\left( {\frac{{f_{l} }}{{f_{co} }}} \right)} \right]f_{co}$	$\varepsilon_{cc} = \left[ {1 + 20\left( {\frac{{f_{l} }}{{f_{co} }}} \right)} \right]\varepsilon_{co}$	Circular
Teng and Lam 2002^50^	$f_{cc} = \left[ {1 + 2\left( {\frac{{f_{l} }}{{f_{co} }}} \right)} \right]f_{co}$	$\varepsilon_{cc} = \left[ {2 + 15\left( {\frac{{f_{l} }}{{f_{co} }}} \right)} \right]\varepsilon_{co}$	Circular
Lu and Hsu 2006^51^	$f_{cc} = \left[ {1 + 4\left( {\frac{{f_{l} }}{{f_{co} }}} \right)} \right]f_{co}$	$\varepsilon_{cc} = \left[ {1 + 19.21\left( {\frac{{f_{l} }}{{f_{co} }}} \right)} \right]\varepsilon_{co}$	Circular
Legeron and Paultre 2003^52^	$f_{cc} = \left[ {1 + 2.4\left( {\frac{{f_{l} }}{{f_{co} }}} \right)^{0.7} } \right]f_{co}$	$\varepsilon_{cc} = \left[ {1 + 35\left( {\frac{{f_{l} }}{{f_{co} }}} \right)^{1.2} } \right]\varepsilon_{co}$	Circular, square, and rectangular
Triantafillou et al. 2006^53^	$f_{cc} = \left[ {1 + 2.79\left( {\frac{{f_{l} }}{{f_{co} }}} \right)} \right]f_{co}$	$\varepsilon_{cc} = \left[ {\varepsilon_{co} + 0.082\left( {\frac{{f_{l} }}{{f_{co} }}} \right)} \right]$	Circular, square, and rectangular
Akiyama et al. 2010^54^	$f_{cc} = \left[ {1 + 2.4\left( {\frac{{f_{l} }}{{f_{co} }}} \right)^{0.647} } \right]f_{co}$	$\varepsilon_{cc} = \left[ {\varepsilon_{co} + 0.0766\left( {\frac{{f_{l} }}{{f_{co} }}} \right)} \right]$	Circular, square, and rectangular
Shehata et al. 2002^55^	$f_{cc} = \left[ {1 + 0.85\left( {\frac{{f_{l} }}{{f_{co} }}} \right)} \right]f_{co}$	$\varepsilon_{cc} = \left[ {1 + 13.5\left( {\frac{{f_{l} }}{{f_{co} }}} \right)} \right]\varepsilon_{co}$	Square
	$f_{cc} = \left[ {1 + 0.7\left( {\frac{{f_{l} }}{{f_{co} }}} \right)} \right]f_{co}$	$\varepsilon_{cc} = \left[ {1 + 12.4\left( {\frac{{f_{l} }}{{f_{co} }}} \right)} \right]\varepsilon_{co}$	Rectangular
Cusson and Paultre 1995^56^	$f_{cc} = \left[ {1 + 2.1\left( {\frac{{f_{l} }}{{f_{co} }}} \right)^{0.7} } \right]f_{co}$	$\varepsilon_{cc} = \left[ {\varepsilon_{co} + 0.21\left( {\frac{{f_{l} }}{{f_{co} }}} \right)^{1.7} } \right]$	Square
			Rectangular

Confined and unconfined concrete compressive strengths are denoted by $f_{cc}$ and $f_{co}$, respectively, coefficient $k_{1}$ is confinement effectiveness factor, and $f_{l}$ is the lateral confining pressure which can be linked to the strength and thickness of CFRP by formulas given below:

**For circular concrete specimens**

$$f_{l} = \frac{{2f_{T} t}}{D}$$

$f_{T}$ describes the CFRP tensile strength in the hoop direction. The thickness of CFRP confinement is defined by *t*, and the core concrete diameter is specified by *D*.

**For square concrete specimens**

$$f_{l} = \frac{{2f_{T} tk_{s} }}{D}$$

$k_{s}$ is the shape factor, defined as the ratio of effective confinement area to concrete cross-sectional area. The following equation, as given by ACI318^57^, can be used to calculate this shape factor:

$$k_{s} = 1 - \left[ {\frac{{\left( {b - 2R_{c} } \right)^{2} + \left( {h - 2R_{c} } \right)^{2} }}{{3A_{g} }}} \right]$$

$A_{g}$ is the concrete’s cross-sectional area, which can be calculated using the equation below:

$$A_{g} = bh - \left( {4 - \pi } \right)R_{{c^{2} }}$$

The equivalent circular specimen is defined as a specimen with a CFRP volumetric ratio that is similar to the original rectangular specimen. The equivalent diameter of the circular specimen can be found using the equation below, where *b* is for width, and *h* is for depth of the rectangular or square section:

$$D = \frac{2bh}{{b + h}}$$

## Comparison of experimental and analytical results

Table 6 compares the results of existing theoretical models with the experimental results. From the comparison, it can be seen that the theoretical estimated strength of circular concrete specimens for most of the models is greater than the experimental results for a single layer of CFRP confinement. However, for a double layer of CFRP confinement, most of the strength models yield results closer to experimental findings. Results of strength models given by Teng and Lam^50^ and Triantafillou et al.^53^ are closer to experimental results of CFRP confined single layer circular concrete specimens, and results of strength models given by Lu and Hsu^51^, Legeron and Paultre^52^ and Akiyama et al.^54^ are almost equal to the experimental results of double layered CFRP confined circular concrete specimens. For theoretical predictions of square concrete specimens, most of the strength models have higher theoretical predictions than experimental results for both single and double CFRP confinement. Results of strength models given by Shehata et al.^55^ and Triantafillou et al.^53^ are closer to the experimental results of square concrete specimens having a single layer of CFRP confinement, and the model given by Cusson and Paultre^56^ is approximately equal to the experimental result of CFRP circular concrete specimens with a double layer. For theoretical predictions of rectangular concrete specimens, all strength model’s prediction is lower than that of experimental findings for both single and double layers of CFRP.

**Table 6:** Comparison of experimental and analytical results for circular concrete specimens.

Study	Specimen	$f_{cc}$(Theoretical) MPa	$f_{cc}$(Experimental) MPa	$\varepsilon_{cc}$(Theoretical)	$\varepsilon_{cc}$(Experimental)	Shape
Candappa et al. 2001^49^	C20E-WCL1	54.49	45	0.489	0.21	Circular
	C20E-WCLII	72.97	64	0.818	0.72	
Teng and Lam 2002^50^	C20E-WCL1	43.40	45	0.567	0.21	Circular
	C20E-WCLII	50.79	64	0.813	0.72	
Lu and Hsu 2006^51^	C20E-WCL1	50.79	45	0.476	0.21	Circular
	C20E-WCLII	65.57	64	0.791	0.72	
Legeron and Paultre 2003^52^	C20E-WCL1	53.56	45	0.524	0.21	Circular
	C20E-WCLII	64.53	64	0.998	0.72	
Triantafillou et al. 2006^53^	C20E-WCL1	46.32	45	0.168	0.21	Circular
	C20E-WCLII	56.63	64	0.177	0.72	
Akiyama et al. 2010^54^	C20E-WCL1	54.83	45	0.168	0.21	Circular
	C20E-WCLII	65.48	64	0.176	0.72	
Shehata et al. 2002^55^	S20E-WCL1	30.05	32	0.189	0.19	Square
	S20E-WCLII	31.10	38	0.258	0.21	
Legeron and Paultre 2003^52^	S20E-WCL1	36.63	32	0.215	0.19	Square
	S20E-WCLII	41.40	38	0.338	0.21	
Triantafillou et al. 2006^53^	S20E-WCL1	32.44	32	0.123	0.19	Square
	S20E-WCLII	35.88	38	0.127	0.21	
Akiyama et al. 2010^54^	S20E-WCL1	37.57	32	0.123	0.19	Square
	S20E-WCLII	42.42	38	0.126	0.21	
Cusson and Paultre 1995^56^	S20E-WCL1	35.68	32	0.121	0.19	Square
	S20E-WCLII	39.85	38	0.123	0.21	
Shehata et al. 2002^55^	R20E-WCL1	21.46	32	0.189	0.17	Rectangular
	R20E-WCLII	21.92	34	0.258	0.14	
Legeron and Paultre 2003^52^	R20E-WCL1	25.45	32	0.215	0.17	Rectangular
	R20E-WCLII	28.22	34	0.338	0.14	
Triantafillou et al. 2006^53^	R20E-WCL1	22.83	32	0.123	0.17	Rectangular
	R20E-WCLII	24.65	34	0.127	0.14	
Akiyama et al. 2010^54^	R20E-WCL1	26.08	32	0.123	0.17	Rectangular
	R20E-WCLII	28.95	34	0.126	0.14	
Cusson and Paultre 1995^56^	R20E-WCL1	24.89	32	0.121	0.17	Rectangular
	R20E-WCLII	27.32	34	0.123	0.14	

Table 6 also compares the results of existing theoretical models of strain with the experimental results. A comparison of results concluded that some of the theoretical results are on the higher side, and some are on the lower side as compared to the experimental results. Shehata et al.’s^55^ theoretical results for square and rectangular concrete specimens with a single layer are significantly equal to experimental results. Theoretical prediction of strain models for rectangular concrete specimens having a double layer, as suggested by Triantafillou et al.^53^, Akiyama et al.^54^ and Cusson and Paultre^56^ are equal to experimental observations. From the theoretical and experimental results comparison, it can be concluded that these model estimations of results are not very accurate, and more extensive analysis is required for concrete specimens that contain E-waste and have CFRP confinement so that better prediction of results can be achieved. The inaccuracy of these models could be associated with the use of natural coarse aggregates and the mechanical properties of concrete. Most of the models in the literature were developed for the confinement of natural aggregate concrete. The strain of concrete having E-waste, on the other hand, is shown to be lower than that of natural aggregate concrete. As a result, the predictions of existing models (which were developed for natural aggregate concrete) were not accurate for CFRP-confined concrete made with E-waste aggregates. The compressive strength of concrete having E-waste aggregate is less than the theoretical value for a single CFRP confinement layer. This is likely due to the single layer of CFRP's considerable lateral dilatation and lack of confinement. Lateral dilatation in circular samples is restrained when enough confinement is given, such as in the case of a double CFRP confinement, resulting in higher strength and strain of CFRP confined samples than predicted by theoretical models. More research is needed to fully comprehend the process of lateral dilation and CFRP confinement in concrete having E-waste aggregates as a replacement for natural coarse aggregates.

## Conclusions

Based on the experimental work, the following main points can be concluded from this research work:

1. Natural coarse aggregate replacement with E-waste resulted in lower concrete strength and higher failure strain. Compressive strength is reduced by 32.5%, 33.3%, and 37% in circular, square, and rectangular samples, respectively, when 20% coarse aggregates are replaced with E-waste. Although the strength of E-waste concrete is lower than that of concrete made with natural aggregates, there are many positive net benefits of recycled electronic waste compared to the procurement and production of natural aggregates. The use of E-waste aggregates is not only financially beneficial but there are a variety of benefits related to the environment and sustainability as fewer resources would be required to produce the same aggregate material without destroying the natural resources. The use of E-waste could be beneficial to real construction. The E-waste concrete could be considered a controlled low-strength material (CLSM). The CLSM concrete could be effectively used for non-structural purposes such as backfill or road bases. Also, high-strength E-waste concrete could be produced by the use of suitable admixtures, and practical implications could be extended to the structural load-bearing components such as beams and columns.
2. CFRP confinement has a higher impact in the case of circular concrete specimens. Compressive strength is increased by 67% using a single CFRP layer and 137% using two CFRP layers when 20% E-waste coarse aggregates were added to circular samples with CFRP confinement. As a result of stress concentration at sharp corners of square and rectangular samples, CFRP sheet rupture at lower stress than circular geometry, so square and rectangular samples showed less increment in strength by using CFRP confinement.
3. CFRP sheets also enhanced the specimens’ Poisson’s ratio. Because of the greater confinement given by a double CFRP layer, it is more effective than a single layer. Confinement of E-waste concrete specimens using CFRP layers improved the specimens' stiffness, strength, and ductility.
4. Failure patterns reveal that the square and rectangular specimens' asymmetrical geometry causes lateral CFRP to rupture at sharp corners, making them less effective than circular specimens.
5. From theoretical and experimental results comparison, it can be concluded that these model estimations of results are encouraging. However, this study itself is a novel and a preliminary step in this very particular research area, Therefore, for the development of better consensus, further research studies are recommended.

### Recommendations for future research

In future studies, there is a need to explore the efficiency of E-waste aggregates and CFRP composites for large-scale reinforced concrete circular and square columns subjected to lateral cyclic loading. In circular and square columns, the shear span to depth ratio could be varied to investigate different structural behaviors such as shear, flexural shear, and flexural behavior of concrete columns. Further, the effect of lap splices in the plastic hinge region could be studied for concrete columns made with both E-waste and natural aggregates. Also, in future studies, detailed finite element analysis is required for the CFRP-confined large-scale columns.

## Data Availability

The datasets generated during and/or analysed during the current study are available from the corresponding author on reasonable request.
